# A Comprehensive Mathematical Model of Motor Unit Pool Organization, Surface Electromyography, and Force Generation

**DOI:** 10.3389/fphys.2019.00176

**Published:** 2019-03-08

**Authors:** Eike Petersen, Philipp Rostalski

**Affiliations:** Institute for Electrical Engineering in Medicine, University of Lübeck, Lübeck, Germany

**Keywords:** mathematical modeling, electromyography, force generation, motor unit, rate coding, recruitment, action potential, neuromuscular physiology

## Abstract

Neuromuscular physiology is a vibrant research field that has recently seen exciting advances. Previous publications have focused on thorough analyses of particular aspects of neuromuscular physiology, yet an integration of the various novel findings into a single, comprehensive model is missing. In this article, we provide a unified description of a comprehensive mathematical model of surface electromyographic (EMG) measurements and the corresponding force signal in skeletal muscles, both consolidating and extending the results of previous studies regarding various components of the neuromuscular system. The model comprises motor unit (MU) pool organization, recruitment and rate coding, intracellular action potential generation and the resulting EMG measurements, as well as the generated muscular force during voluntary isometric contractions. Mathematically, it consists of a large number of linear PDEs, ODEs, and various stochastic nonlinear relationships, some of which are solved analytically, others numerically. A parameterization of the electrical and mechanical components of the model is proposed that ensures a physiologically meaningful EMG-force relation in the simulated signals, in particular taking the continuous, size-dependent distribution of MU parameters into account. Moreover, a novel nonlinear transformation of the common drive model input is proposed, which ensures that the model force output equals the desired target force. On a physiological level, this corresponds to adjusting the rate coding model to the force generating capabilities of the simulated muscle, while from a control theoretic point of view, this step is equivalent to an exact linearizing transformation of the controlled neuromuscular system. Finally, an alternative analytical formulation of the EMG model is proposed, which renders the physiological meaning of the model more clear and facilitates a mathematical proof that muscle fibers in this model at no point in time represent a net current source or sink. A consistent description of a complete physiological model as presented here, including thorough justification of model component choices, will facilitate the use of these advanced models in future research. Results of a numerical simulation highlight the model's capability to reproduce many physiological effects observed in experimental measurements, and to produce realistic synthetic data that are useful for the validation of signal processing algorithms.

## 1. Introduction

Electromyography (EMG) denotes the measurement of the electrical fields generated by the electrophysiological processes that lead to muscle fiber contraction. EMG is highly relevant for a number of clinical and scientific applications, since it enables monitoring and analysis of a muscle's electromechanical properties and state, both of which would otherwise remain mostly inaccessible. Surface electromyography (sEMG) denotes the noninvasive measurement of electrical muscle activity by means of electrodes placed on the skin surface, as opposed to the traditional measuring method using needle electrodes. For more background information on sEMG, its analysis and many of its applications, refer to, e.g., Merletti and Parker (2004) and Merletti and Farina (2016).

Mathematical models of sEMG are highly useful, on the one hand to advance understanding of the underlying physiological processes, and on the other hand to analyze the sensitivity of sEMG measurements to various physiological and technical parameters, and to test and validate sEMG signal processing algorithms. Over the past decades, researchers have pursued a number of different approaches for the modeling and simulation of different aspects of sEMG measurements. Phenomenological (Hogan and Mann, 1980; Lo Conte et al., 1994; Sinderby et al., 1995; McGill, 2004) as well as physiologically motivated (Fuglevand et al., 1993; Dimitrov and Dimitrova, 1998; Farina and Merletti, 2001; Farina et al., 2004; Dideriksen et al., 2010a,b; Mordhorst et al., 2015) models have been proposed and analyzed. Overviews can be found in Stegeman et al. (2000), McGill (2004), Rodriguez-Falces et al. (2012), and Merletti and Farina (2016).

Many researchers have worked on modeling the electric signal produced by a single contraction of a single muscle fiber, the so-called single fiber action potential (SFAP). Classically, the propagation of the action potential along a contracting muscle fiber has been modeled using simplified dipole, tripole or quadrupole models Fuglevand et al. (1992); Merletti et al. (1999); Merletti and Parker (2004); Plonsey and Barr (2007). A more general model has been proposed by Dimitrov and Dimitrova (1998), and this model has been successfully employed, modified and combined with various other models for the remaining physiological processes in several publications Farina and Merletti (2001); Farina et al. (2004); Wang et al. (2006); Dideriksen et al. (2010a).

In the present article, the SFAP model originally proposed by Dimitrov and Dimitrova (1998), and subsequently extended by Farina and Merletti (2001), is combined with the well-known motor unit (MU) pool organization model of Fuglevand et al. (1993) and the twitch force parameterization used by Raikova and Aladjov (2002). Care is taken in particular to achieve a consistent parameterization of the electrical and the mechanical components of the model, resulting in a realistic EMG-force relationship of the simulated muscle. Recent results regarding the modeling of MU rate coding and recruitment (De Luca and Hostage, 2010) and the variability of the inter-spike intervals (Moritz et al., 2005) are incorporated, and a new model of motor unit firing rates is proposed. Moreover, we propose a novel nonlinear transformation of the common drive input to the muscle, which ensures that the desired muscle output force can directly be used as a model input. This is achieved by adjusting the rate coding model to the force generating capabilities of the simulated muscle and has interesting consequences for the modeling of physiological force control, which will be discussed as well. Finally, an alternative analytical formulation of the SFAP model of Farina and Merletti (2001) is proposed, which clarifies the physiological meaning of the model. Based on this alternative formulation, a proof is provided that in this model the ingoing and outgoing currents along each fiber sum to zero at all times, which is a physiologically plausible property due to the quasi-static behavior of action potential generation (Plonsey and Barr, 2007).

In section 2, all components of the mathematical model are presented briefly, yet completely, and in a unified way. Several mathematical properties of the model are derived, and the above-mentioned alternative formulation of the model of Farina and Merletti (2001) is proposed. Results of numerical simulations based on the model are presented in section 3 and are assessed with respect to their physiological plausibility. Finally, section 4 concludes the article with a discussion of the various improvements introduced in this article. Note that a preliminary version of the mathematical analysis of the sEMG model of Farina and Merletti (2001) presented in section 2.5 has been the subject of a conference publication (Petersen, 2016).

## 2. Mathematical Model

**Figure 1:**
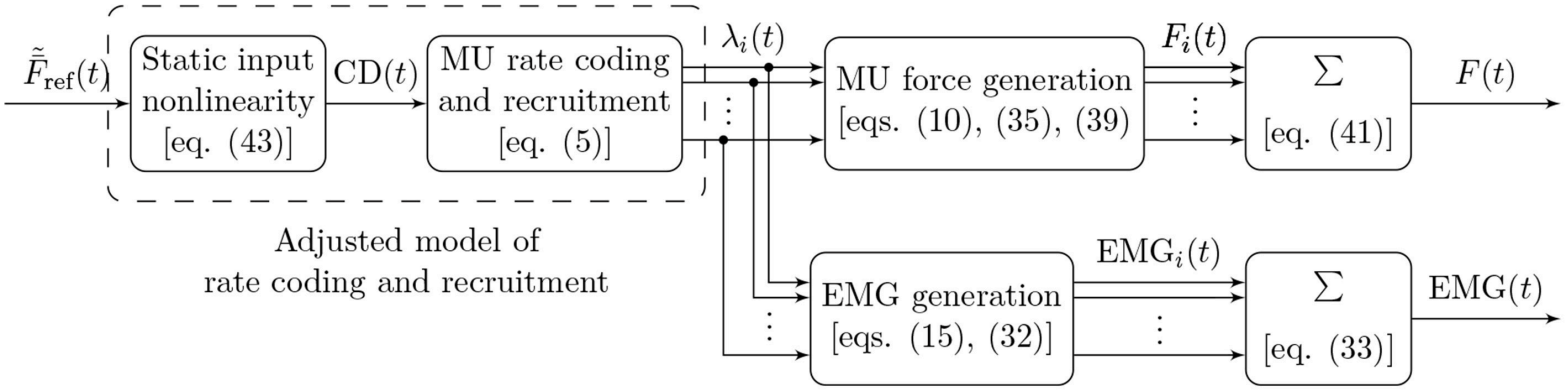
Block diagram illustrating the main components of the proposed model of muscular force generation. The model input is the mean normalized desired muscle force ${\tilde{\bar{F}}}_{\text{ref}}\left(t\right)$ with values in [0, 1], the model outputs are the total generated muscle force *F*(*t*) and the measured EMG signal. Firing rates of individual MUs are denoted by λ_*i*_(*t*), the force contribution of each MU by *F*_*i*_(*t*), and the EMG contribution of each MU by EMG_*i*_(*t*). The common drive input CD(*t*) to the rate coding model is calculated from the desired muscle force by means of a static, nonlinear function, which effectively adjusts the rate coding and recruitment model to the force generation model.

The fundamental functional unit of a skeletal muscle is the motor unit (MU), comprising a motor neuron and the muscle fibers innervated by that neuron. The following sections introduce mathematical models of the electrical and mechanical properties of MUs, as well as their organization in a muscle. Figure 1 shows a graphical summary of the main model components and their interactions and may provide a useful reference for the reader while following along the description of the model.

### 2.1. Motor Unit Pool Structure

Every muscle consists of a number *n* of MUs. Each MU has various mechanical and electrical properties, most of which have been found to be closely related through the *size principle*: MU size as measured by the number of fibers contained in the MU is roughly proportional to force twitch amplitude, EMG twitch amplitude, and recruitment threshold (Henneman, 1957; Henneman et al., 1965; Heckman and Enoka, 2012). This means that larger MUs are only activated at higher levels of desired muscle force compared to smaller units, but they also add larger force and EMG contributions to the muscle output once activated. This orderly recruitment appears to be a result of an increase in input resistance with MU size (Powers and Binder, 2001; Heckman and Enoka, 2012) and seems to remain remarkably stable over a wide range of MU conditions (Heckman and Enoka, 2012). Based on these findings, the classical MU pool model of Fuglevand et al. (1993) that has been reused and extended in numerous studies (Stegeman et al., 2000; Zhou and Rymer, 2004; Keenan and Valero-Cuevas, 2007; Dideriksen et al., 2010a; Robertson, 2014; Potvin and Fuglevand, 2017) describes the MU twitch forces and contraction times as a function of the MU's recruitment threshold. Recent results have shown that various other parameters are also related to MU size, and hence can be considered size principle parameters as well (Heckman and Enoka, 2012; Del Vecchio et al., 2017). In the following, we will describe a size-dependent MU pool parameterization model that is based on the model of Fuglevand et al. (1993), but extended in several important aspects. Currently, the most common assumption regarding the neural excitation of MUs is that all MUs in a muscle receive the same net level of neural input, usually termed the *common drive* to the muscle (De Luca and Erim, 1994; Erim et al., 1996; Piotrkiewicz and Türker, 2017). We therefore formulate our model as a function of the common drive CD(*t*), with 0 ≤ CD(*t*) ≤ 1.

The recruitment thresholds of MUs in a muscle appear to follow a continuous distribution with many MUs attaining a small recruitment threshold, and few large MUs only being recruited at high activation levels (Van Cutsem et al., 1997; Raikova et al., 2007; Heckman and Enoka, 2012). This behavior is captured well by the exponential model proposed by Fuglevand et al. (1993), which assigns the recruitment thresholds

(1) $$\begin{array}{l} {\text{CD}}_{\text{rec}(i)}=\frac{e^{ai}}{100}\text{ with }a=\frac{\ln(100\cdot {\text{CD}}_{\text{full}})}{n} \end{array}$$

to MUs *i* = 1, …, *n*, where CD_rec(*i*)_ denotes the minimum level of common drive at which the MU is recruited, and CD_full_ denotes the point of full recruitment, i.e., the level of common drive at which all MUs are recruited. Alternatively, following the formulation of De Luca and Contessa (2012), the thresholds can also be modeled as

(2) $$\begin{array}{l} {\text{CD}}_{\text{rec}(i)}=\frac{bi}{n}\cdot \frac{e^{ai}}{100}\text{ with }a=\frac{\ln(\frac{100\text{·}{\text{CD}}_{\text{full}}}{b})}{n}, \end{array}$$

where *b* denotes a scaling factor that influences the shape of the distribution. The latter model results in a more gradual slope compared to the first one and has been used by De Luca and Contessa (2012) for modeling the characteristics of the Vastus Lateralis (VL) muscle. The choice between Equations (1) and (2), in general, should be based on the characteristics of the specific muscle under consideration. With the recruitment thresholds set, peak twitch forces are calculated as a linear function of the recruitment thresholds following

(3) $$\begin{array}{l} P_{i}=P_{1}+\frac{{\text{CD}}_{\text{rec}(i)}-{\text{CD}}_{\text{rec}(1)}}{{\text{CD}}_{\text{full}}-{\text{CD}}_{\text{rec}(1)}}\text{ · }(P_{n}-P_{1}) \end{array}$$

as proposed by Contessa and De Luca (2013), where the twitch peak range (*P*_*n*_/*P*_1_) is typically large, e.g., *P*_*n*_/*P*_1_ = 130 for the First Dorsal Interosseus (FDI) muscle (Fleshman et al., 1981; Contessa and De Luca, 2013).

Equivalently to Equation (3), Fuglevand et al. (1993) modeled the number η of innervated muscle fibers, which appears to be the main factor influencing MU twitch force (Totosy de Zepetnek et al., 1992), directly proportional to the peak twitch force and hence also to the recruitment threshold. This direct proportionality, however, neglects the increase in fiber diameter from small to large MUs, which also contributes to the overall rise in MU twitch force (Burke, 1981; Kernell, 2006), since specific fiber tension (tension/area) does not change significantly (Lucas et al., 1987; Heckman and Enoka, 2012). The fiber diameter also determines, among others, action potential amplitude (Hakansson, 1956; Nandedkar and Stålberg, 1983), and electrical twitch conduction velocity (Hakansson, 1956; Nandedkar and Stålberg, 1983; Sadoyama et al., 1988). We thus consider both peak fiber twitch force and single fiber action potential (SFAP) amplitude to be size principle parameters as well, with the former being proportional to the square of the recruitment threshold CD_rec_ (since it is related to fiber *area*) and the latter directly proportional to CD_rec_ (since it is related to fiber *diameter*). Finally, a fivefold range in contraction speed has been found from the slowest to the fastest MU (Burke, 1981; Fleshman et al., 1981) and there is also a slight negative correlation of time to peak force with the recruitment threshold (Van Cutsem et al., 1997), while the electrical twitch conduction velocity *v* correlates positively with the recruitment threshold (Del Vecchio et al., 2017). We hence consider the electrical conduction velocity *v* and the force twitch model parameters *T*_EMD_, *T*_ri_, and *T*_hr_ (see section 2.7) to be linearly related to the recruitment threshold as well.

Fuglevand et al. (1993) modeled the relationships between parameter values deterministically, but experimental evidence demonstrates that these relations are of a highly stochastic nature (Heckman and Enoka, 2012). As did several previous studies (Contessa and De Luca, 2013; Robertson, 2014; Al Harrach et al., 2017), we thus draw parameter values for each MU at random from a stochastic distribution. The main novelty of the MU pool part of our model is that the distribution from which each property is drawn depends continuously on the MU's size. Previous modeling studies have followed a different path in assigning each MU a particular *MU type* or each fiber a *fiber type* and then drawing parameter values at random from a parameter distribution associated with this type (Robertson, 2014; Al Harrach et al., 2017), or drawing the parameters of all MUs from the same, static distribution (Contessa and De Luca, 2013). It has been argued, however, that both MU and fiber properties follow a *continuous* distribution that does not support a distinction between discrete types (Fuglevand et al., 1993; Enoka and Fuglevand, 2001; Heckman and Enoka, 2012; Potvin and Fuglevand, 2017). For this reason, we decided to model both MU and fiber properties as continuously distributed quantities. As proposed by Contessa and De Luca (2013), we use Weibull distributions for the parameter values. Summarizing, the MU pool model presented above describes the electrical and mechanical parameters of individual MUs as a stochastic, continuous function of MU size.

One final difference between our model and previous models regards the model input: while previous studies have always described recruitment thresholds as a function of the muscle force *F*(*t*) (Fuglevand et al., 1993; De Luca and Contessa, 2012), we define them as a function of the common drive CD(*t*) instead. This change is necessary since the generated muscle force depends on all properties of the MU pool, and not only on the recruitment thresholds. Finding a model parameterization that fixes the MU recruitment thresholds at particular levels of muscle force is, hence, a hard problem that requires careful adjustment of all model components. It has to the authors' knowledge not been solved so far. The relationship between the common drive and the generated muscle force, as well as an alternative solution to the problem of adjusting model components to each other, will be discussed in more detail in section 2.8.

### 2.2. Geometrical Distribution of Motor Units and Muscle Fibers

Muscle fibers belonging to the same motor unit spread over a territory that may span a large portion of the muscle cross section (Buchthal et al., 1957; Burke, 1981; Trontelj et al., 2004). The territories of the different MUs overlap, leading to an interweaving of fibers belonging to multiple MUs (Buchthal et al., 1957; Trontelj et al., 2004). Buchthal et al. (1957) have found motor unit territories to attain an irregular round shape, whence we propose the use of an elliptic model for the MU cross sections. With the elliptic axis ratio being fixed, the MU cross-sectional area—and thereby the axes lengths—are calculated by dividing the number of innervated fibers *η* by the desired MU fiber density *ρ* (fibers/area):

(4) $$\begin{array}{l} A=\frac{\eta }{\rho }. \end{array}$$

The midpoints of all MUs are then distributed uniformly over the muscle cross section. Note that without further assumptions, the above model directly leads to overlapping regions between MUs, which is a desirable feature, as noted above. Finally, fibers belonging to the MU are then again distributed uniformly inside the elliptic MU cross section. This model is equivalent to the propositions of Fuglevand et al. (1993), except for the means of reducing fiber density variability described next, and the fact that they used circular MU territory shapes as opposed to the more flexible elliptic shape proposed here. It is advisable to divide the muscle cross section into *M* parts of equal size, and then distribute *n*/*M* MUs uniformly in each part, to avoid an unrealistically high variability of the fiber density due to the random MU placement. Alternatively, MU centers could also be placed using an optimization algorithm such as the one proposed by Carriou et al. (2016b), which sequentially places MUs at the position that maximizes the distance to the already placed MUs.

A crucial decision when modeling random MU placement concerns the treatment of muscle boundaries. Those parts of MU territories that exceed the muscle territory must be cut off, and the question remains how to account for this loss in MU territory. Several approaches to solving the problem are conceivable (Rodriguez-Falces et al., 2012; Carriou et al., 2016b):

1. All fibers belonging to the MU are placed in the remaining parts of the MU territory. This approach leads to an increase in the fiber density of boundary MUs, and hence also in the overall fiber density toward the muscle boundaries.
2. The number of fibers innervated by the MU is reduced proportionally. This approach keeps the assigned fiber density constant, but reduces the number of innervated fibers and thus the size of boundary MUs.
3. The axes lengths of the elliptic MU region are adjusted in such a way as to keep the MU area at the desired value in spite of the cut-off. This approach keeps the number of fibers and the fiber density at the desired values but likely leads to strongly increased overall fiber density toward the muscle center, due to many adjusted MU regions overlapping there.
4. MUs with territories exceeding the muscle territory are rejected completely. This approach obviously removes the need for MU property adjustments, but without further modifications leads to reduced overall fiber density close to the muscle boundaries (Carriou et al., 2016b).

There are advantages and disadvantages to each approach, and it does not yet seem to be clear if one of the proposed approaches is generally superior to the others, or which approximates reality best (Rodriguez-Falces et al., 2012). However, muscle fiber diameters appear to be approximately constant throughout a muscle (Johnson et al., 1973; Schnetzer et al., 2001), whence a constant fiber density throughout the muscle cross section seems desirable. To this end, the second of the above approaches has been pursued here.

Reducing only the number of fibers in MUs close to the muscle boundary without adjusting the other MU parameters as well would disturb the relationship between the electrical and mechanical properties of these MUs. Hence, the recruitment threshold CD_rec(*i*)_, the peak twitch force *P*_*i*_ and the electrical twitch conduction velocity *v* were recomputed. Note that this, in turn, distorts the exponential distribution of MU parameters described by Equations (1) and (2). This distortion, however, was considered less grave than a disturbed electromechanical relationship in a significant number of MUs. Finally, to prevent unphysiologically sharp IAP generation and extinction artifacts, we randomize both innervation zone placement as well as fiber end placement, as described previously (Merletti et al., 1999; Carriou et al., 2016b).

An alternative model of MU placement has been proposed by Navallas et al. (2010). They explicitly consider the optimization problem of minimizing the variability of muscle fiber density throughout the muscle, regardless of variances in MU fiber density and while maintaining the exponential relationship (1). Their model has recently been extended (Robertson and Johnston, 2017) to account for the physiologically observed regionalized MU placement (Elder et al., 1982), something that has not been but could be considered in our model. While all of these are desirable properties of a MU placement algorithm, the much simpler division of the muscle region into separate parts and random placement proposed above also reduces the variability of the fiber density. MU regionalization could be implemented using this same division into separate parts; and the distortion to the exponential relationship (1) when using this algorithm has been found to be rather small in practice, see section 3. In summary, both the placement algorithms of Navallas et al. (2010) and the one proposed here represent viable modeling choices, with the former being preferable if the particular influence of different aspects of MU geometry is of interest, and the latter being a much simpler algorithm.

### 2.3. Firing Rates

**Figure 2:**
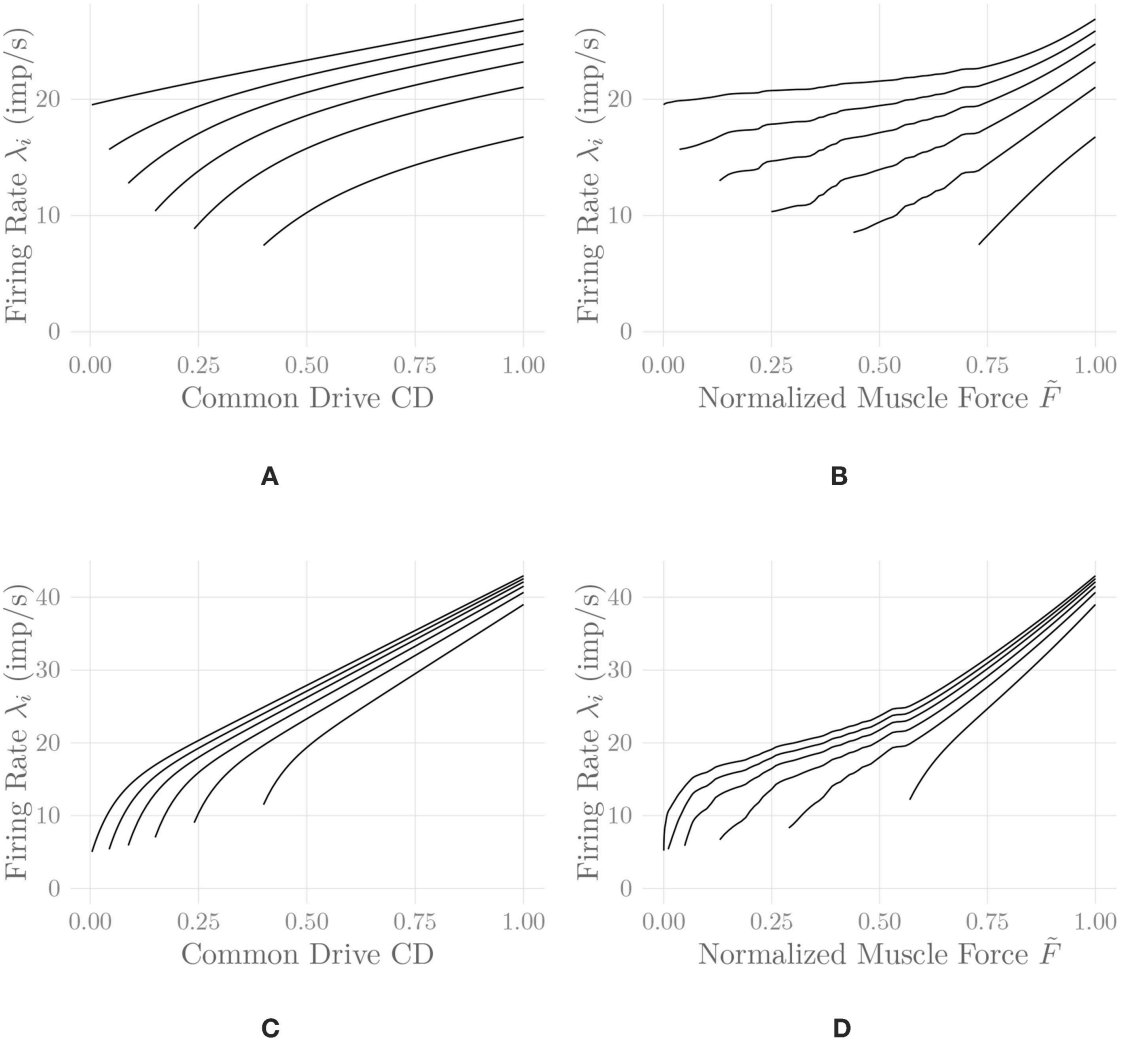
Firing rates of every tenth MU in one belly of one of the simulated *recti*, following Equation (5) **(A,B)** and Equation (6) **(C,D)**. Firing rates are shown as a function of the common drive CD(*t*) to the muscle **(A,C)** and as a function of the normalized muscle output force $\tilde{F}$ **(B,D)**. The point of full recruitment was set to CD_full_ = 0.6. Note that the application of the proposed nonlinear input transformation $\text{CD}={\tilde{F}}^{-1}\left({\tilde{F}}_{\text{ref}}\right)$ has shifted the point of full recruitment to a much higher force level. This shift and the noticeable upwards bend at high activation levels are a result of adjusting the rate coding model to the force generating capabilities of the muscle.

The firing rate λ_*i*_(*t*) of a motor unit denotes the frequency at which its motoneuron discharges, thereby generating both EMG and force twitches in its innervated muscle fibers. Together with the force and EMG twitch amplitudes of each MU, the firing rates are the primary determinant of the generated muscle force and surface EMG. Following the experimental results of Milner-Brown et al. (1973), firing rates have traditionally been modeled as a linear function of excitatory drive (Fuglevand et al., 1993; Erim et al., 1996). Recently, De Luca and Hostage (2010) have proposed a linear-exponential firing rate model based on new experimental findings. In this model, the firing rate of a MU with recruitment threshold CD_rec(*i*)_ is given by

(5) $$\begin{array}{l} \text{λ}(\text{CD}(t);\;{\text{CD}}_{\text{rec}(i)})=C_{4}\text{·CD}(t)+(C_{3}-C_{1}e^{-\text{CD}(t)/C_{2}})\text{·}{\text{CD}}_{\text{rec}(i)}+C_{5}, \end{array}$$

where 0 ≤ CD(*t*) ≤ 1 denotes the current level of common drive to the muscle, and the *C*_*j*_, *j* = 1, …, 5 are constant shape parameters. For example values of the shape parameters for different muscles, refer to De Luca and Hostage (2010). Figure 2A shows exemplary firing rate characteristics obtained using Equation (5).

This model reproduces many phenomena observed experimentally, such as the *onion-skin phenomenon*: MUs recruited first appear to attain a higher firing rate than those MUs recruited later throughout the whole contraction (De Luca and Erim, 1994; Erim et al., 1996). Interestingly, however, the model also differs from previous results and models in several ways. Firstly, the model does not feature the increased slope of the firing rate characteristics at excitation levels exceeding the point CD_full_ of full recruitment which has been observed by De Luca and Erim (1994). Secondly, in the observations and the model of De Luca and Hostage (2010), the initial firing rates of motor units are *declining* with increasing recruitment threshold, while in the earlier study of Erim et al. (1996), they are *increasing*. Thirdly, the firing rates, especially of the smaller motor units, appear unusually large in the low activity range. These high rates may result from the limitation of the regression data, from which the model was derived, to the range from 20 to 100% MVC, as has been noted by the authors in a subsequent corrigendum (De Luca and Hostage, 2012). While the parameters in Equation (5) of course can be tuned to follow new findings or represent other muscles, the shape of the characteristics cannot be altered arbitrarily due to the small number of model parameters. Finally, other researchers have questioned the validity of the EMG decomposition approach employed by De Luca and Hostage (2010) to obtain the firing rates of individual MUs (De Luca and Nawab, 2011; Farina and Enoka, 2011; Farina et al., 2015), a fact that warrants caution when reusing results obtained using this decomposition approach.

To facilitate the inclusion of the various observations discussed above into a single model, and since the model of De Luca and Hostage (2010) cannot be adjusted in all of these regards, we propose the following, novel model of MU firing rates:

(6) $$\begin{array}{l} \text{λ}(\text{CD}(t);{\text{CD}}_{\text{rec}(i)})=-C_{1}\cdot (C_{2}-\text{CD}(t))\cdot {\text{CD}}_{\text{rec}(i)}+C_{3}\cdot \text{CD}(t)\\ \;+C_{4}-(C_{5}-C_{6}\cdot {\text{CD}}_{\text{rec}(i)})\cdot e^{-\frac{\text{CD}(t)-{\text{CD}}_{\text{rec}(i)}}{C_{7}}}\text{​}. \end{array}$$

Figure 2C shows exemplary firing rate characteristics obtained using this model. This model (with suitably chosen parameter values *C*_*j*_, *j* = 1, …, 7) fulfills the following requirements:

- An initial, steep slope of the characteristics is followed by a flatter, linear region, and the transition between the two regions is smooth. Neither the models of Fuglevand et al. (1993) nor Erim et al. (1996) are smooth. Also, the model of Fuglevand et al. (1993) does not feature the initial phase with steeper slopes.
- At each activation level CD(*t*), the characteristics fulfill the *onion skin property* dλ(CD; CD_rec(*i*)_)/dCD_rec(*i*)_ < 0.
- The slope dλ(CD = CD_rec(*i*)_; CD_rec(*i*)_)/dCD_rec(*i*)_ of the initial firing rates can be freely adjusted to either positive or negative values. This is not the case for the model of De Luca and Hostage (2010).
- The degree of convergence of the firing rate characteristics of earlier and later recruited MUs can be freely adjusted by modifying *C*_2_. This is not possible using the model of De Luca and Hostage (2010). The importance of this issue has been discussed in detail by Fuglevand et al. (1993).

Note that as for the recruitment thresholds, we define the firing rates as a function of the common drive, as did Fuglevand et al. (1993) and unlike De Luca and Hostage (2010). The generated muscle force depends on all model parameters, and hence firing rates at a particular level of muscle force must be chosen in accordance with the force twitch parameters of the involved MUs. We will address this issue in section 2.8 by introducing a suitably defined procedure for calculating the common drive input CD(*t*) to the rate coding model as a function of the desired muscle force level ${\tilde{F}}_{\text{ref}}$, thereby adjusting the rate coding model to the force generating capacity of the model. One benefit of the nonlinear input transformation proposed in section 2.8 is that it effectively results in an increased slope of the firing rate characteristics at excitation levels exceeding the point of full recruitment CD_full_, a desirable feature as was mentioned above.

### 2.4. Firing Instants

Given the instant *t*_*i*(*j*−1)_ of the (*j* − 1)th firing of MU *i* and the time course of the MU's firing rate λ_*i*_, the next firing instant *t*_*ij*_ can be calculated. To model the stochastic distribution of the inter-spike intervals, these are assumed to follow a normal distribution with a coefficient of variation that decreases with increasing activation, following

(7) $$\begin{array}{l} c_{v,i}(\text{CD}(t))=10+20e^{-(\text{CD}(t)-{\text{CD}}_{\text{rec}(i)})/2.5}, \end{array}$$

as proposed by Moritz et al. (2005). The *j*^th^ inter-spike interval ISI_*j*_ then is drawn from

(8) $$\begin{array}{l} {\text{ISI}}_{j}\sim N\left({\text{ISI}}_{j}^{*},\;c_{v,i}(CD(t_{ij}^{*}))\cdot {\text{ISI}}_{j}^{*}\right), \end{array}$$

where the mean $t_{ij}^{*}$ of the time of the next firing event and the corresponding mean inter-spike interval ${\text{ISI}}_{j}^{*}$ are obtained by solving

(9) $$\begin{array}{l} t_{ij}^{*}-t_{i(j-1)}=\frac{1}{{\text{λ}}_{i}(\text{CD}(t_{ij}^{*}))}={\text{ISI}}_{j}^{*} \end{array}$$

for $t_{ij}^{*}$ as proposed by Fuglevand et al. (1993). The time *t*_*ij*_ of the *j*^th^ firing event is finally calculated as

(10) $$\begin{array}{l} t_{ij}=t_{i(j-1)}+{\text{ISI}}_{j}. \end{array}$$

Note that several distributions other than the normal distribution have been proposed for modeling the distribution of the inter-spike intervals (Jones et al., 2002; Barry et al., 2007). For reasons of simplicity, and as the influence of the shape of the distribution of the inter-spike intervals on the overall EMG and force signals found by Barry et al. (2007) appeared to be rather negligible, a normal distribution is used in the simulation described in section 3.

Equation (7) represents a purely phenomenological model of force variability that reproduces the experimentally observed decrease in coefficient of force variation with increasing activation (Moritz et al., 2005). This phenomenon appears to be the result of different physiological processes, with synaptic noise being the main contributor to the coefficient of variation at low activation levels and oscillations in the descending neural drive explaining most of the variation at higher levels of activation (Dideriksen et al., 2012). Since the model described in this article is purely feed-forward, i.e., no physiological feedback or force control are considered, a phenomenological model of force variability is a reasonable choice. If, however, the model presented here was to be included in a more complete model including force control based on sensory feedback, one should strive to reproduce the experimentally observed variations in force variability through synaptic noise and the intrinsic properties of a force feedback controller such as the one recently proposed by Dideriksen et al. (2017).

One aspect of firing instant calculations that has to the authors' knowledge not been addressed before is the fact that firing instants will occur at arbitrary times between two sampled instants. Simulation studies so far have assumed the set of firing instants *t*_*ij*_ to be a subset of the set of sampling instants (Fuglevand et al., 1993), which is a simplifying assumption that may introduce an artificial distortion in the simulated signal. In our model, we hence allow firing instants to occur at arbitrary times, and not just at sampling instants. Note that for implementing this, it is necessary to calculate differently shifted versions of both the force and EMG twitches of each MU at each firing instant. For the force twitch model presented in section 2.7 an analytical functional relationship is available, for which a time shift can be realized by simply evaluating this function at different input values. Regarding the EMG twitch model described in section 2.6, a description of the Fourier spectrum of the EMG twitch is provided which can be evaluated numerically, and which is then inversely transformed to obtain the EMG twitch in time. Here, a time shift can be realized efficiently in the Fourier domain before applying the inverse transform.

### 2.5. Intracellular Action Potential Propagation

The propagation of an intracellular action potential (IAP) from the neuromuscular junction (NMJ) of a muscle fiber along both directions toward the two fiber ends can be modeled by representing the actively firing fiber by a distributed current source and sink. In the model initially proposed by Dimitrov and Dimitrova (1998), this distributed fiber membrane current source $\hat{ı}\left(z,t\right)$ is composed of two propagating wavefronts and localized contributions at the NMJ and the two fiber ends. These localized contributions model the IAP generation and extinction process. In the formulation of Farina and Merletti (2001), the model reads

(11) $$\begin{array}{l} \hat{ı}\;(z,t)=\frac{\text{d}}{\text{d}z}[\psi \;(z-z_{i}-vt)\;p_{1}(z)-\psi \;(-z+z_{i}-vt)\;p_{2}(z)]. \end{array}$$

Here, *z* denotes the spatial variable along the muscle fiber, *z*_*i*_ the location of the NMJ, *v* the IAP's propagation velocity, and

(12) $$\begin{array}{l} p_{1}\left(z\right)=H\left(z-z_{i}\right)-H\left(z-\left(z_{i}+L_{1}\right)\right)\text{ and}\\ p_{2}\left(z\right)=H\left(z-\left(z_{i}-L_{2}\right)\right)-H\left(z-z_{i}\right) \end{array}$$

the characteristic functions of the two fiber halves, where *L*_1_ and *L*_2_ are the distances between the innervation zone and the right and left tendon, respectively. The propagation velocity *v*, which has been found to depend on the MU's firing rate (Nishizono et al., 1989) among other factors, is assumed constant throughout the whole simulation for each MU in this study to simplify calculations. For an efficient method to simulate different propagation velocities, refer to Dideriksen et al. (2010b). Moreover,

(13) $$\begin{array}{l} \psi (z)=\frac{\text{d}}{\text{d}z}V_{m}(-z), \end{array}$$

denotes the voltage gradient across the fiber membrane along the fiber axis, where the function *V*_*m*_(*z*) prescribes a model for the trans-fiber membrane voltage wave shape and can be chosen arbitrarily to match simulated or measured data. For details on the significance of *V*_*m*_(*z*), refer to Plonsey and Barr (2007). Here, the analytical model function

(14) $$\begin{array}{l} V_{m}(z\;[\text{mm}])=\{\begin{array}{ll} D_{1}z^{3}e^{-z}+D_{2}, & \text{if }z>0\\ D_{2}, & \text{otherwise} \end{array} \end{array}$$

with *D*_1_ = 96 mV mm^−3^ and *D*_2_ = −90 mV will be used, as originally proposed by Rosenfalck (1969) and as has been done in previous studies (Farina and Merletti, 2001; Carriou et al., 2016a).

The IAP model in Equation (11) can be shown to be equivalent to choosing

(15) $$\begin{array}{l} \hat{ı}(z,t)=\text{GEN}(t)\;\delta \;\left(z-z_{i}\right)\;+\psi^{\prime }\left(z-z_{i}-vt\right)p_{1}\left(z\right)\\ \;+{\text{EOF}}_{1}(t)\;\delta \left(z-z_{i}-L_{1}\right)+\psi^{\prime }\left(-z+z_{i}-vt\right)p_{2}\left(z\right)\\ \;+{\text{EOF}}_{2}(t)\;\delta \left(z-z_{i}+L_{2}\right), \end{array}$$

with the Dirac distribution *δ*, the end-of-fiber components

(16) $$\begin{array}{l} {\text{EOF}}_{1}(t)=-\psi \left(L_{1}-vt\right) \end{array}$$

and

(17) $$\begin{array}{l} {\text{EOF}}_{2}(t)=-\psi \left(L_{2}-vt\right), \end{array}$$

and the potential generation component

(18) $$\begin{array}{l} \text{GEN}(t)=2\;\psi \left(-vt\right). \end{array}$$

Here, again, the end-of-fiber components describe the IAP extinction process at the fiber ends, and the potential generation component models the influence of IAP generation at the innervation zone on the membrane current.

This formulation renders—to the authors' opinion—the structure of the model more clear, by explicitly distinguishing between propagating and non-propagating signal components, and by revealing the non-smoothness of the resulting distributed current source, the latter following from the presence of the stationary Dirac distributions at the two fiber ends and the location of the innervation zone. The equivalence of the two formulations of the model is summarized in the following lemma, the proof of which is given in the Appendix.

Lemma 1. *The expressions given in Equations (11) and (15) to (18) are equivalent, assuming that ψ* ∈ $C^{\infty }$^1^.

In order to achieve a smooth current source model as opposed to the discontinuous model proposed by Farina and Merletti (2001) and analyzed above, Carriou et al. (2016a), inspired by earlier works by Merletti et al. (1999), have suggested the use of smooth Tukey window functions *w*_1/2_(*z*) instead of the characteristic functions *p*_1/2_(*z*), which results in the progressive generation and extinction of action potentials. In their study, they set the window smoothness parameter to α = 0.1, where α = 0 would result in the characteristic functions specified in Equation (12). However, while appearing physiologically reasonable, the influence of this modification on the resulting EMG signal and the EMG-force relationship has been found to be limited (Carriou et al., 2016a). Note that a formulation similar to Equations (15)– (18) can easily be obtained if smooth window functions are used instead of the characteristic functions in Equation (11), since in this case all derivatives are defined in the classical sense [assuming smoothness of *ψ*(*z*)].

Interestingly, one can show that both IAP models ensure

(19) $$\begin{array}{l} \overset{\infty }{\underset{-\infty }{\int^{\text{​}}}}\hat{ı}\;(z,\;t)\text{ d}z=0\;\forall \;t, \end{array}$$

which means that the sum of all incoming and outgoing currents along each fiber is zero at all times. This property is well motivated by physiology, considering that the invoked electrodynamical processes can be regarded as quasi-static (Plonsey and Barr, 2007), and it is also in accordance with the predictions of the standard Hodgkin-Huxley model for action potential propagation (Kleinpenning et al., 1990). For the discontinuous model, it is precisely a result of the presence of the three Dirac distributions in Equation (15), as these have the combined effect of collecting all remaining currents exerted by the intermediate fiber sections due to their higher potential. This result is the subject of the following lemma, the proof of which again is deferred to the Appendix.

Lemma 2. *For compactly supported ψ*(*z*), *the IAP model given in Equations (15) to (18) [or equivalently, Equation (11)] yields a formulation of* $\hat{ı}\left(z,t\right)$ *that satisfies the condition (19)*^2^. *This also holds true if the characteristic functions* *p*_1_(*z*), *p*_2_(*z*) *are replaced by smooth window functions* *w*_1_(*z*), *w*_2_(*z*) *which fulfill*

(20) $$\begin{array}{l} w_{1}(z_{i}+L_{1})=w_{1}(z_{i})=w_{2}(z_{i})=w_{2}(z_{i}-L_{2})=0 \end{array}$$

*and which yield a product ψ*(*z*)*w*_1/2_(*z*) *that is differentiable with an integrable derivative*.

The Tukey window function employed in Carriou et al. (2016a) and Al Harrach et al. (2017) falls into the class of window functions supported by the above lemma.

### 2.6. EMG Measurements

Biological tissues can be considered volume conductors (Plonsey and Barr, 2007). The existence of an electric field implies the existence of electric currents traveling through the tissue, and vice versa^3^. Due to the comparably low rate of change of physiological systems, it is justified (Plonsey and Barr, 2007) to assume these time-varying electric fields to behave as if they were static at each instant of time, whence they are called *quasi-static*. This assumption amounts to a neglection of the capacitive properties of the tissues. Accordingly, as for static fields, the electric field in a physiological volume conductor is considered equal to the negative gradient of a scalar potential *φ*, namely,

(21) $$\begin{array}{l} \vec{E}=-\nabla \varphi . \end{array}$$

By Ohm's law, the current density (current per unit of cross-sectional area) in a volume conductor is proportional to the electric field, that is,

(22) $$\begin{array}{l} \vec{J}=\sigma \vec{E}=-\sigma \nabla \varphi , \end{array}$$

where σ denotes the conductivity of the medium. Defining a distributed current density source *I* throughout the region of interest, the divergence of the current density is constrained by

(23) $$\begin{array}{l} \nabla \cdot \vec{J}=I. \end{array}$$

Combining Equations (22) and (23) and assuming a homogeneous, isotropic medium yields Poisson's equation for the diffusion of the potential, namely,

(24) $$\begin{array}{l} \Delta \varphi =-\frac{I}{\sigma }. \end{array}$$

In the following, the electric field generated by point sources in planar tissue layers will be considered as a model for flat and large muscles, such as the *recti abdominis* simulated in section 3. The muscle layer is assumed to be infinitely extended and planar, and to be covered by an infinitely extended planar layer of fat and an infinitely extended planar layer of skin. Muscle tissue is considered anisotropic to reflect the difference in conductivity between currents along the muscle fiber axis and currents across the muscle fiber axis, whereas fat and skin tissue are considered isotropic. Muscle fibers are assumed to run along the *z* direction, with the *x* and *z* dimensions spanning the skin plane, and the *y* dimension being orthogonal to the skin plane, positive vectors pointing outwards.

The geometrical set-up described above has been analyzed by Farina and Rainoldi (1999). For a point source of strength Î located at (0, *y*_0_, 0), the authors derive the 2-D spatial Fourier transform of the resulting potential distribution at the skin surface to be

(25) $$\begin{array}{l} \Phi (\hat{I},\omega_{x},\omega_{z};y_{0})=\frac{2\hat{I}}{\sigma_{m,p}}e^{-\omega_{ya}|y_{0}|}\\ \;\cdot \frac{1}{(1+r_{c})\;\cosh\left(\omega_{y}^{+}\right)\;\nu \left(\omega_{y}^{+}\right)+(1-r_{c})\;\cosh\left(\omega_{y}^{-}\right)\;\nu \left(\omega_{y}^{-}\right)}, \end{array}$$

with the abbreviations

(26) $$\begin{array}{l} \omega_{y}^{+}=\omega_{y}(d_{f}+d_{s}),\;\omega_{y}^{-}=\omega_{y}(d_{f}-d_{s}),\\ \omega_{y}=\sqrt{\omega_{x}^{2}+\omega_{z}^{2}},\;\omega_{ya}=\sqrt{\omega_{x}^{2}+r_{a}\omega_{z}^{2}} \end{array}$$

and

(27) $$\begin{array}{l} \text{ν}(s)=\omega_{ya}+sr_{m}\tanh(s), \end{array}$$

where ω_*x*_ = 2π*f*_*x*_ and ω_*z*_ = 2π*f*_*z*_ denote the spatial angular frequencies in the *x* and *z* directions, respectively. The coefficients

(28) $$\begin{array}{l} r_{c}=\frac{\sigma_{s}}{\sigma_{f}},\;r_{m}=\frac{\sigma_{f}}{\sigma_{m,p}},\text{ and }r_{a}=\frac{\sigma_{m,f}}{\sigma_{m,p}} \end{array}$$

specify ratios of the different tissue conductivities. Finally, *y*_0_ denotes the depth of the point source in the muscular tissue, *d*_*f*_ the thickness of the fat layer and *d*_*s*_ the thickness of the skin layer.

Equation (25) directly yields an analytic description of the 2-D spatial transfer function of the volume conductor *via*

(29) $$\begin{array}{l} H_{\text{vc}}(\omega_{x},\omega_{z};\;y_{0})=\frac{1}{\hat{I}}\cdot \text{Φ}(\hat{I},\omega_{x},\omega_{z};\;y_{0}). \end{array}$$

EMG measurements are usually taken differentially between a set of electrodes. Consider a regular grid of *R* × *S* electrodes with interelectrode distances *d*_*x*_ and *d*_*z*_, respectively, where *R* = *R*_*a*_ + *R*_*b*_ + 1 and *S* = *S*_*a*_ + *S*_*b*_ + 1. The variables subscripted by *a* and *b* denote the number of electrodes on the two sides of an arbitrarily chosen reference electrode. The grid is assumed to be aligned parallel to the *z* axis. Assigning weights ζ_*kℓ*_ to the electrodes and assuming all electrodes to attain the same transfer function, the (spatial) transfer function from a given surface potential distribution to the potential measured by such an electrode configuration at each point on the surface is given by (Farina and Merletti, 2001)

(30) $$\begin{array}{l} H_{\text{ec}}\left(\omega_{x},\omega_{z}\right)=\sum_{k=-R_{a}}^{R_{b}}\sum_{\text{ℓ}=-S_{a}}^{S_{b}}\zeta_{k\text{ℓ}}e^{-j\omega_{x}kd_{x}}e^{-j\omega_{z}\text{ℓ}d_{z}}. \end{array}$$

For the transfer function *H*_ele_ of a single electrode, various model assumptions can be made as well. For examples and details, please refer to, e.g., Merletti and Parker (2004).

Concatenating the spatial transfer functions *H*_vc_ of the volume conductor, *H*_ec_ of the electrode configuration and *H*_ele_ of the electrodes themselves, the global transfer function of the combined system emerges as

(31) $$\begin{array}{l} H_{\text{glo}}\left(\omega_{x},\omega_{z};y\right)=H_{\text{vc}}\left(\omega_{x},\omega_{z};y\right)\cdot H_{\text{ele}}\left(\omega_{x},\omega_{z}\right)\cdot H_{\text{ec}}\left(\omega_{x},\omega_{z}\right). \end{array}$$

**Figure 3:**
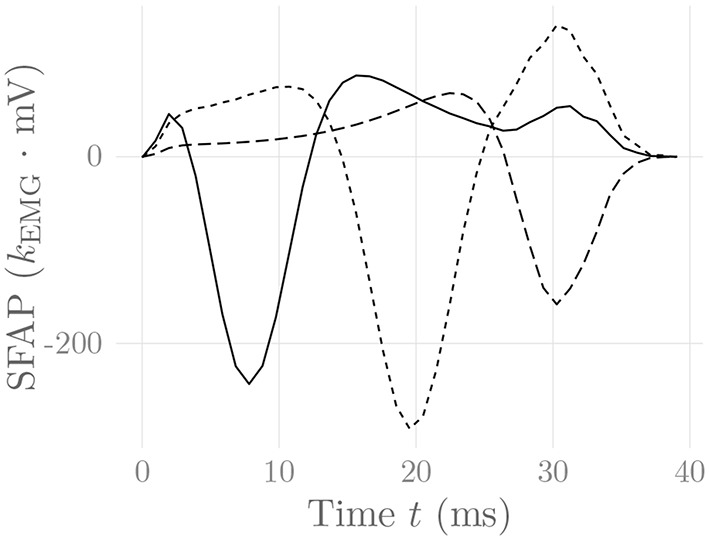
Simulated SFAPs evoked by a single firing muscle fiber as detected by three surface electrodes positioned close to the NMJ (solid), in between the NMJ and the fiber end (short dashes) and above the fiber end (long dashes). The constant *k*_EMG_ represents an arbitrary, static EMG scaling factor.

From this, the 2-D potential distribution on the skin surface as measured by electrode configurations consisting of electrodes with transfer function *H*_ec_ and *H*_ele_, respectively, and positioned at (*x, z*), can generally be calculated as

(32) $$\begin{array}{l} \varphi (x,z,t)=\int_{\mathbb{R}}\left(i\left(x,y,z,t\right)\underset{\left(x,z\right)}{*}h_{\text{glo}}\left(x,z;y\right)\right)\text{ d}y\\ \;=\int_{\mathbb{R}}F_{xz}^{-1}\left\{i\left(\omega_{x},y,\omega_{z},t\right)\cdot H_{\text{glo}}\left(\omega_{x},\omega_{z};y\right)\right\}\text{ d}y \end{array}$$

where *i*(ω_*x*_, *y*, ω_*z*_, *t*) = $F_{xz}$ {*i*(*x, y, z, t*)} is the 2-D Fourier transform of the current density source *i*(*x, y, z, t*), and *_(*x, z*)_ denotes 2-dimensional convolution in the *x* and *z* variables. For a particular electrode (configuration) location on the skin surface and a muscle fiber following a straight line parallel to the skin surface, Equation (32) simplifies, and the resulting single-fiber action potential SFAP(*t*) = φ(*t*) can be calculated numerically (Farina and Merletti, 2001; Petersen, 2015; Carriou et al., 2016a). One can prove that in this case the integration kernel only has removable singularities, which ensures the convergence of a numerical integration scheme (Petersen, 2015). Figure 3 shows exemplary SFAPs resulting from the evaluation of Equation (32) for such fibers using nested numerical integration schemes. Note that while the above derivation has been performed for the case of planar volume conductors, Farina et al. (2004) have derived a similar model for cylindrical volume conductors, which is much more appropriate for the simulation of limb muscles.

The numerical solution of the (simplified version of) Equation (32) is computationally moderately expensive (Carriou et al., 2016a), refer to section 3 for a discussion of computation times. Fortunately, this only has to be done once for each fiber before the actual simulation, to calculate the SFAPs of all fibers. During the simulation, multiple shifted versions of these SFAPs are then superposed to generate the actual EMG measurement

(33) $$\begin{array}{l} \text{EMG}\left(t\right)=\sum_{i=1}^{n}\sum_{j=1}^{N_{i}}{\text{MUAP}}_{i}\left(t-t_{ij}\right), \end{array}$$

where *n* denotes the number of MUs, *N*_*i*_ the number of firing events of that MU, and

(34) $$\begin{array}{l} {\text{MUAP}}_{i}\left(t\right)=\sum_{j=1}^{\eta_{i}}{\text{SFAP}}_{ij}\left(t\right) \end{array}$$

the motor unit action potential, which is obtained by summing over the SFAPs of all muscle fibers *j* belonging to MU *i*. For a discussion of how to evaluate Equation (32) efficiently especially in the HD-sEMG case, refer to Carriou et al. (2016a). The simulated SFAPs correctly reproduce the dependency of the SFAP shape on the relative position of the recording electrode and the depth of the muscle fiber, as well as the distinction between propagating and localized signal components at the NMJ and the two fiber ends. In particular, the SFAPs display the experimentally observed end-of-fiber extinction components.

### 2.7. Force Twitches

Each MUAP generates a corresponding force contribution, denoted as a force twitch *f*_*i*_(*t*). Most previously proposed muscle models (Fuglevand et al., 1993; Dideriksen et al., 2010a) have employed the force twitch parameterization of Milner-Brown et al. (1973), in which a force twitch is entirely described by its twitch rise time *T*_ri_ and its peak twitch force *P*. This model, however, does not allow setting the half relaxation time *T*_hr_ independently of *T*_ri_, which is essential for modeling different muscle fiber types (refer to Figure 1.2 in Merletti and Parker, 2004, for example). For this reason, we propose the use of a different model. Raikova and Aladjov (2002) introduced an additional degree of freedom to obtain the following force twitch model of increased expressiveness:

(35) $$\begin{array}{l} f_{i}\left(t\right)=\{\begin{array}{ll} 0, & \;t<T_{\text{EMD},i},\\ p_{i}\cdot {\left(t-T_{\text{EMD},i}\right)}^{m}\cdot e^{-\kappa \left(t-T_{\text{EMD},i}\right)}, & \;t\geq T_{\text{EMD},i} \end{array} \end{array}$$

with

(36) $$\begin{array}{l} p_{i}=P_{i}\cdot e^{-\kappa T_{\text{ri,}i}\left(\log T_{\text{ri,}i}-1\right)}, \end{array}$$

(37) $$\begin{array}{l} m=\kappa \cdot T_{\text{ri},i}, \end{array}$$

and

(38) $$\begin{array}{l} \kappa =\frac{\log2}{T_{\text{hr,}i}-T_{\text{ri},i}\log\left(T_{\text{hr},i}/T_{\text{ri},i}\right)-T_{\text{ri},i}}. \end{array}$$

**Figure 4:**
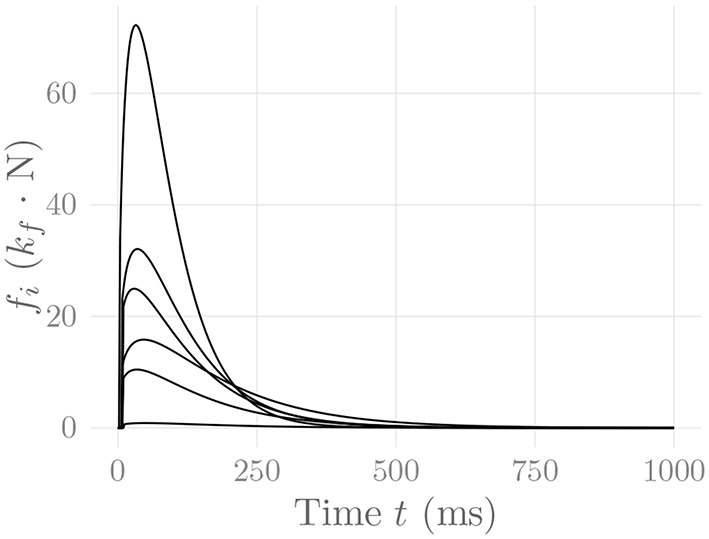
Force twitches generated by every tenth MU of the muscle simulated in section 3. These are the same MUs for which the rate coding characteristics are shown in Figure 2. The constant *k*_*f*_ represents an arbitrary, static force scaling factor.

This model satisfies *f*_*i*_(*T*_ri,*i*_) = *P*_*i*_, and *f*_*i*_(*T*_hr,*i*_) = *P*_*i*_/2. The parameter *T*_EMD_ denotes the electromechanical delay between the onset of electrical and mechanical activity of the motor unit. The three parameters *T*_ri_, *T*_hr_ and *T*_EMD_ are sampled from a Weibull distribution for each MU, as proposed (for the former two) by Contessa and De Luca (2013). Figure 4 shows some exemplary force twitches generated by this model. Note that fixing *m* = 1 and freely selecting *p*_*i*_ and κ results in the force model of Milner-Brown et al. (1973).

Kernell et al. (1983) have found experimentally that there is a nonlinear relationship between generated isometric muscle force and firing rates of MUs: At high firing rates, the force twitch amplitude decreases. The proposed model includes this nonlinear relationship by scaling individual force twitches in the impulse train of a particular MU by a factor

(39) $$\begin{array}{l} g_{ij}\left({\tilde{\text{λ}}}_{ij}\right)=\{\begin{array}{ll} 1 & 0\leq {\tilde{\text{λ}}}_{ij}\leq 0.4\\ \frac{0.4}{{\tilde{\text{λ}}}_{ij}\left(1-\gamma_{2}\right)}\left(1-\gamma_{2}e^{\frac{\left(0.4-{\tilde{\text{λ}}}_{ij}\right)}{\gamma_{1}}}\right) & {\tilde{\text{λ}}}_{ij}>0.4, \end{array} \end{array}$$

with γ_2_ and γ_1_ constant muscle parameters, as proposed by Contessa and De Luca (2013). Here, *g*_*ij*_ denotes the gain factor assigned to the *j*th firing of MU *i*, and ${\tilde{\lambda }}_{ij}$ is the normalized instantaneous firing rate at that firing event:

(40) $$\begin{array}{l} {\tilde{\text{λ}}}_{ij}=T_{\text{ri},i}/{\text{ISI}}_{j}. \end{array}$$

Finally, the total force generated by a muscle is calculated as the superposition

(41) $$\begin{array}{l} F\left(t\right)=\sum_{i=1}^{n}F_{i}\left(t\right)=\sum_{i=1}^{n}\sum_{j=1}^{N_{i}}f_{i}\left(t-t_{ij}\right) \end{array}$$

of the individual force twitches of all MUs, with *n* the number of MUs in the muscle, *F*_*i*_(*t*) the force contribution of MU *i* over time, *N*_*i*_ the number of firing events of MU *i*, and *t*_*ij*_ the *j*^th^ firing instant of MU *i*, calculated following Equation (10).

### 2.8. Excitation-Force Relationship and Physiological Force Control

Motor unit firing rate models such as the model of De Luca and Hostage (2010) described in section 2.3 usually define the firing rate as a function of the desired normalized muscle force ${\tilde{F}}_{\text{ref}}=F_{\text{ref}}/F_{\text{max}}$, i.e., they choose the common drive input as $\text{CD}\equiv {\tilde{F}}_{\text{ref}}$. This choice is a consequence of the fact that these models are derived from experimental measurements of MU firing rates at different muscle force levels. Defining the firing rates λ_*i*_ of all MUs (e.g., as described in section 2.3) as a function of the force target ${\tilde{F}}_{\text{ref}}$ and also defining the force generating properties of these MUs (e.g., as described in section 2.7) uniquely determines the generated muscle force *F*, see Figure 1. It is, however, by no means guaranteed that the generated muscle force *F* will be equal to the force reference *F*_ref_ that has been used as an input to the firing rate model. In other words, the generated muscle force output does not match the desired muscle force. This discrepancy is a consequence of the fact that the rate coding/recruitment model is not adjusted to the force generating capacity of the simulated muscle (or vice versa). Both have been defined individually, in parts based on physiological measurements, but they need to be adapted to each other. Note that this is a general problem that every EMG-force model needs to solve.

Dideriksen et al. (2010b) and Venugopal et al. (2017) solved this issue by introducing a simple PID controller for adjusting the model input, such that the error between desired and actual force output is minimized. This method corresponds to the introduction of an artificial feedback loop in Figure 1. In their model, parameters change over time (simulating fatigue), rendering the introduction of such a feedback loop an elegant solution to the problem of time-varying input-output consistency. Contessa and De Luca (2013) introduced a similar force feedback loop, although they implemented the feedback in an offline instead of online fashion, updating the input signal in hindsight and re-running the simulation if the force output deviated too strongly from the desired output. While all three articles (Dideriksen et al., 2010b; Contessa and De Luca, 2013; Venugopal et al., 2017) include some remarks on physiological feedback processes, neither model was meant to replicate properties of actual physiological feedback control, but rather to account for the fact that the rate coding and force generation model components had not been adjusted to each other. One significant drawback of this method is that the feedback loop unpredictably distorts the characteristics of the assumed rate coding model, effectively leading to a different model being used in simulation than the one that has been initially described. Finally, note that many researchers have worked on understanding and modeling actual physiological feedback control (Wolpert and Ghahramani, 2000; Todorov and Jordan, 2002; Dideriksen et al., 2017).

While there certainly is a feedback element in physiological force control, we consider it reasonable that this feedback is mainly required to compensate for external disturbances and changing muscle properties, not to account for a static input-output inconsistency of the neuromuscular system. For these reasons, we propose to model physiological force control as the combined action of two separate components:

1. a *feed-forward component* that adjusts the rate coding and recruitment model to the force generation model such that in the undisturbed and static case, the muscle's force output equals the desired target force, and
2. a *feedback component* that processes sensory information to counteract external disturbances and changing muscles properties due to muscular fatigue and changes in muscle geometry.

**Figure 5:**
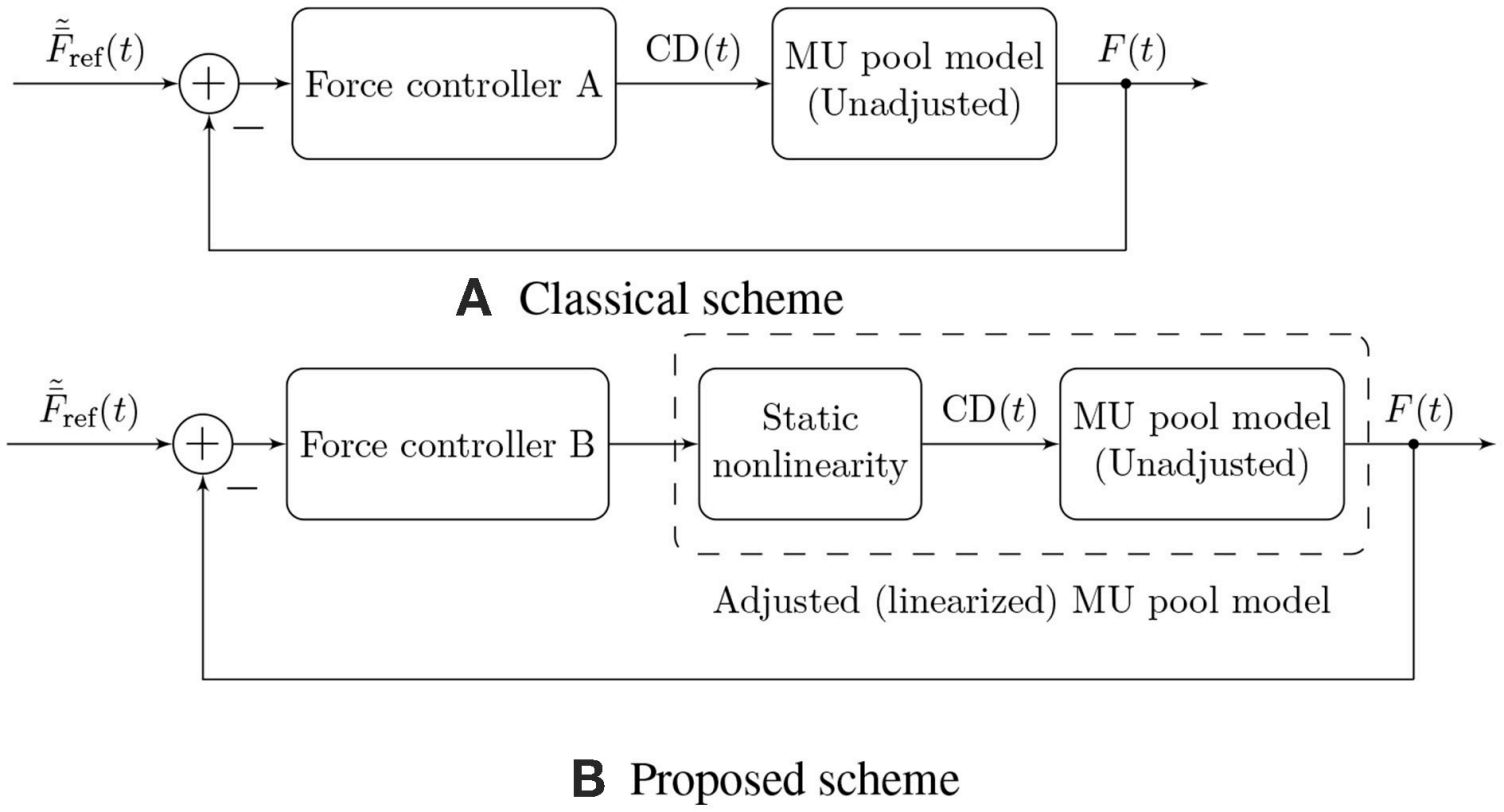
Illustration of two physiological force control schemes. Traditionally, authors have followed approach **(A)**, in which the controller needs to compensate for the nonlinearity of the muscular force generation model in addition to external disturbances and time-varying parameters (Dideriksen et al., 2010b; Contessa and De Luca, 2013; Venugopal et al., 2017). This nonlinearity is not a physiological property but rather results from the fact that the rate coding model is not adjusted to the force generating capacity of the simulated muscle (or vice versa). We propose the use of control scheme **(B)**, in which a static nonlinearity is used to adjust the rate coding model to the force generation model, yielding an adjusted, exactly linearized model of muscular force generation and leaving the feedback controller only with the task of compensating for external disturbances and time-varying model parameters.

Figure 5 illustrates the proposed force control scheme, as opposed to the scheme used traditionally (Dideriksen et al., 2010b; Contessa and De Luca, 2013; Venugopal et al., 2017). In this article, we focus on describing a comprehensive feed-forward model of muscular EMG and force generation, and hence we only consider the first of the two components here. Our approach to the modeling of the feed-forward component is based on a simple, static input nonlinearity that is applied equally to all MUs in the muscle.

The general idea is as follows: denoting the desired normalized muscle force by ${\tilde{F}}_{\text{ref}}\in \left[0,1\right]$, our aim is to choose the common drive $\text{CD}\left({\tilde{F}}_{\text{ref}}\right)\in \left[0,1\right]$ to the MU pool such that

(42) $$\begin{array}{l} \tilde{F}\left(\text{CD}\left({\tilde{F}}_{\text{ref}}\right)\right)={\tilde{F}}_{\text{ref}}\;\forall {\tilde{F}}_{\text{ref}}\in [0,1], \end{array}$$

where $\tilde{F}\left(\text{CD}\right)$ is the normalized generated force output. To achieve this aim, we evaluate $\tilde{F}\left(\text{CD}\right)$ for all CD ∈ [0, 1], i.e., the normalized generated muscle force as a (nonlinear) function of the common drive. Utilizing the inverse

(43) $$\begin{array}{l} \text{CD}={\tilde{F}}^{-1}\left({\tilde{F}}_{\text{ref}}\right) \end{array}$$

as the common drive input to the firing rate model then yields a simulation model that satisfies condition (42). Next, we will derive an analytical expression for the nonlinear function $\tilde{F}\left(\text{CD}\right)$ that can be evaluated efficiently.

Averaging over individual firing events, the mean generated muscle force as a function of the common drive is given by

(44) $$\begin{array}{l} \bar{F}\left(\text{CD}\right)=\sum_{i=1}^{n}g_{i}\left({\text{λ}}_{i}\left(\text{CD}\right)\right)\cdot \Omega_{i}\cdot {\text{λ}}_{i}\left(\text{CD}\right), \end{array}$$

where

(45) $$\begin{array}{l} \Omega_{i}=\int_{0}^{\infty }f_{i}\left(t\right)\text{ d}t=p_{i}\cdot \kappa^{-m-1}\cdot \Gamma \left(m+1\right), \end{array}$$

with Γ(*x*) the Gamma function, denotes the total impulse generated by a single force twitch *f*_*i*_(*t*) of MU *i*, *g*_*i*_ denotes the nonlinear force gain factor defined in Equation (39), and $\bar{x}$ in general denotes the mean over time of a variable *x*. By evaluating

(46) $$\begin{array}{l} \tilde{\bar{F}}=\frac{1}{{\bar{F}}_{\max}}\cdot \sum_{i=1}^{n}g\left({\text{λ}}_{i}\left(\text{CD}\right)\right)\cdot \Omega_{i}\cdot {\text{λ}}_{i}\left(\text{CD}\right) \end{array}$$

for different values of the common drive CD, one can determine $\tilde{\bar{F}}\left(\text{CD}\right)$. Employing $\text{CD}={\tilde{\bar{F}}}^{-1}\left({\tilde{\bar{F}}}_{\text{ref}}\right)$ as the common drive input to the firing rate model then yields a simulation model that satisfies

(47) $$\begin{array}{l} \frac{\bar{F}\left(\text{CD}\left({\tilde{\bar{F}}}_{\text{ref}}\right)\right)}{{\bar{F}}_{\max}}={\tilde{F}}_{\text{ref}}\;\forall {\tilde{\bar{F}}}_{\text{ref}}\in [0,1]. \end{array}$$

Condition (47) ensures that while there may be differences between desired and generated muscle force at individual time instants due to the stochastic nature of the firing instants (see section 2.4), the two forces agree on average.

The model described above for the feed-forward component of physiological force control corresponds to the static input nonlinearity also depicted in Figures 1 and 5 determining the common drive input to the rate coding and recruitment model as a function of the target force level. The introduction of this static input nonlinearity represents an adjustment of the firing rate model to the force generation model, since the rate coding model now no longer receives ${\tilde{F}}_{\text{ref}}$ as an input, but rather its nonlinear transformation ${\tilde{F}}^{-1}\left({\tilde{F}}_{\text{ref}}\right)$. The consequences of this model adjustment will be assessed in the following section, and we will discuss its plausibility. Note that from a control theoretic point of view, the approach presented here represents an exact linearizing transformation of the control plant, as indicated in Figure 5B.

## 3. Simulation Results

Using the mathematical model presented in the previous section, a numerical simulation of the rectus abdominis muscle has been implemented as a test scenario, using the R programming language (R Core Team, 2017). In total, 300 MUs have been simulated, each consisting of between 30 and 150 muscle fibers, organized into three separate muscle bellies of each of the two recti (left and right). Single differential detection was assumed for the EMG signals, with the two electrodes placed on the linea alba between the second and third belly. Parameter values obtained from physiological measurements of the rectus abdominis have been chosen for those parameters where they were available (Delp et al., 2001; Rankin et al., 2006; Teyhen et al., 2012). A series of constant-force isometric contractions has been simulated at progressively increasing force levels in steps of 5%MVC up to 100%MVC. Each step lasted 4 s, adding to a total simulation time of 80 s at a simulated sampling frequency of 1024 Hz. Using parallel processing on an Intel Core i7-6700K 4.0 GHz quad core processor with 32 GB of memory, the generation of one such muscle and the calculation of the corresponding sEMG twitch shapes took approximately 3.5 h, while the subsequent simulation of the described contraction using this muscle model took (on a single core) approximately 1 d. In order to assess the effect of the inherent randomness of the MU placement algorithm, the muscle generation procedure and the subsequent simulation each have been executed five times. The simulated signals are available online at the Dryad data repository (Petersen and Rostalski, 2019).

Figure 2 shows a comparison of the two discussed rate coding models with and without the static input nonlinearity introduced in section 2.8. It can be observed that the input nonlinearity introduces an increase in the slope of the firing rate curves of all MUs at high activation levels in both models. This is in perfect agreement with the experimental findings of Erim et al. (1996) and also makes sense from an intuitive point of view: Once most MUs are recruited, firing rates must increase faster than before in order to achieve an increase in muscle force output. It hence appears that the introduction of this static input nonlinearity, calculated by considering all model parameters simultaneously, increases the degree of similarity between experimental observations and models of rate coding and recruitment. The non-smoothness of the adjusted rate coding characteristics is a consequence of the same non-smoothness in the original activation-force relationship, due to new MUs being recruited at discrete levels of excitation. Accordingly, the non-smoothness of the adjusted rate coding characteristics is required to obtain a smooth input-output force relationship. Note that for lack of better data, we employed rate coding model parameter values similar to the ones described by De Luca and Hostage (2010) for the vastus lateralis (VL) muscle, values that have been selected to match isometric measurements between 20%MVC and 100%MVC (De Luca and Hostage, 2010). This may be a reason for the relatively high initial firing rates observable in Figure 2, as compared to physiological measurements (Erim et al., 1996). The following further results have all been obtained using the model of De Luca and Hostage (2010), since no experimentally justified parameters values for our newly proposed rate coding model are currently available.

**Figure 6:**
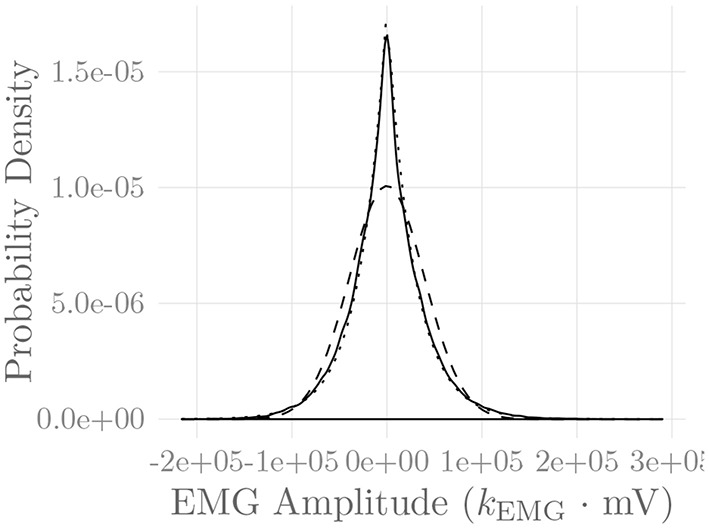
Amplitude distribution of the simulated sEMG signal over the course of a series of isometric contractions at progressively increasing activation levels (solid line). Superimposed are a Gaussian (dashed line) and a Laplacian (dotted line) distribution. The constant *k*_EMG_ represents an arbitrary, static EMG scaling factor.

**Figure 7:**
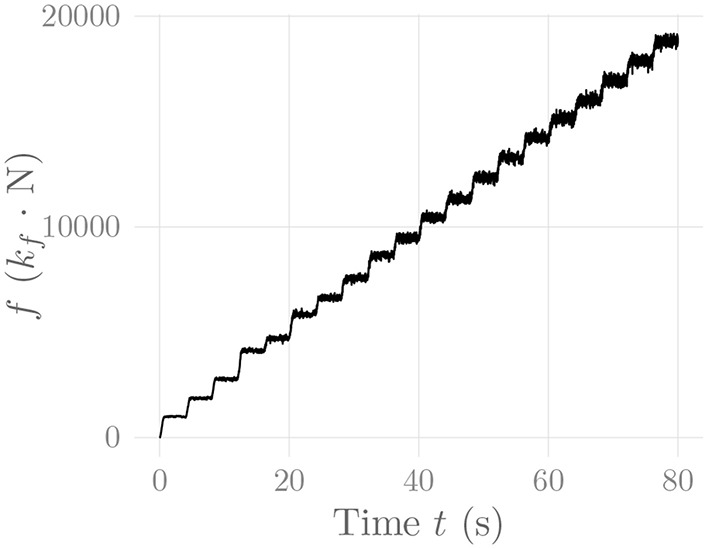
Simulated force signal of the simulated *recti* during a series of isometric contractions. The constant *k*_*f*_ represents an arbitrary, static force scaling factor.

**Figure 8:**
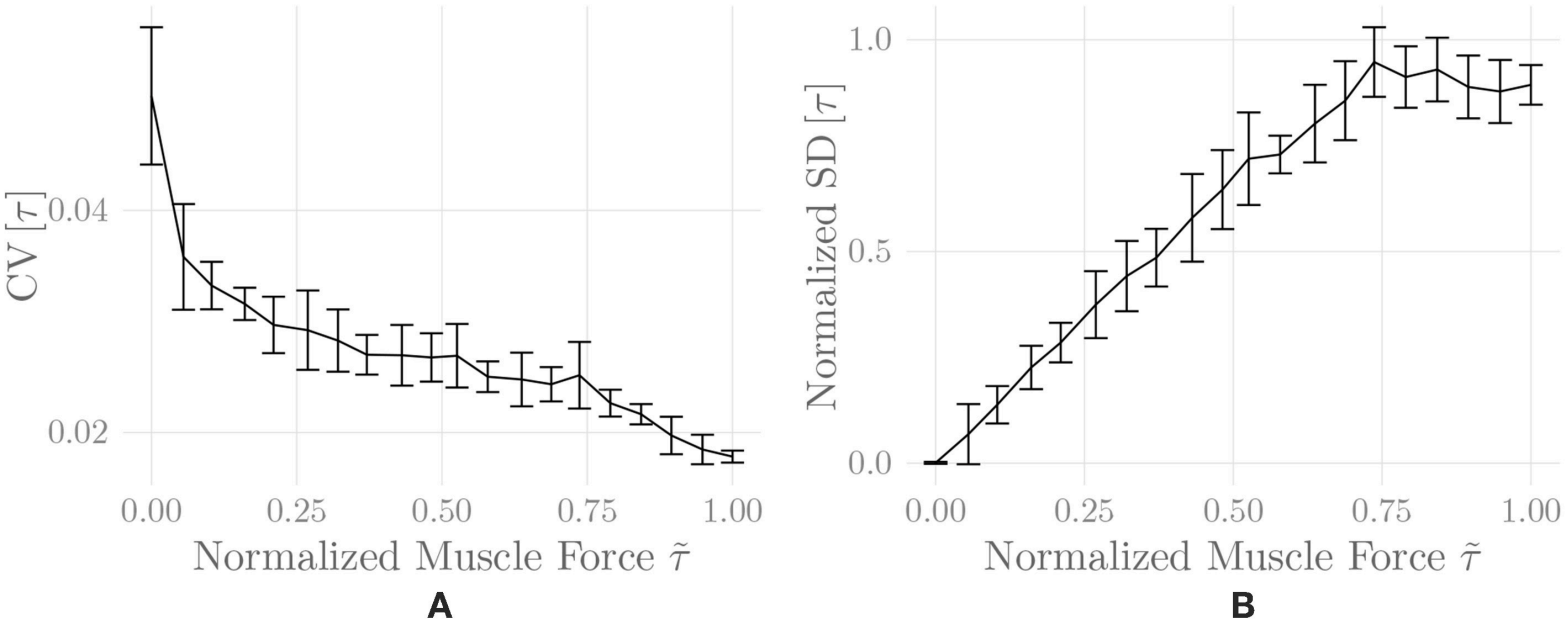
Coefficient of variation **(A)** and normalized standard deviation **(B)** of the simulated force signal of one belly of the simulated *recti* during a series of isometric contractions as a function of muscle activation. Shown are mean ± standard deviation of these values over two simulation runs (including repeated MU placement).

**Figure 9:**
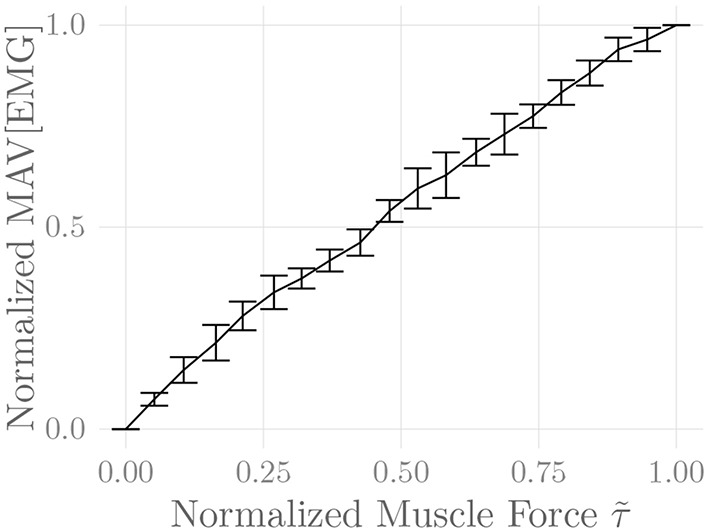
Steady-state sEMG-Force relation of the simulated *recti abdominis* over the course of the simulated constant-force isometric contractions. The first 250 ms of each contraction were discarded, and mean force and mean absolute value (MAV) of the EMG signal were calculated over the remainder of the contraction. Both values were normalized to their minima and maxima over all contractions. Shown are the means of these values over five simulation runs (including repeated MU placement).

Figure 6 shows the amplitude distribution of the simulated EMG signal, which resembles a smoothed Laplacian distribution. This type of distribution has been reported previously for the amplitude distributions of real measurement signals (Clancy and Hogan, 1999). Figure 7 displays the simulated series of isometric contractions; Figure 8 shows the coefficient of variation and the standard deviation of the simulated force signal at different levels of muscle activation. Both force variability graphs resemble those reported by Barry et al. (2007) for experimental measurements of index finger force steadiness. Finally, Figure 9 shows the sEMG-Force relationship calculated over the simulated series of isometric contractions.

## 4. Discussion and Conclusion

In this article, a comprehensive, feed-forward model of surface EMG and force generation during voluntary isometric contractions in skeletal muscles has been described. The model consists of a large number of linear PDEs, ODEs, and various static, sometimes stochastic nonlinear relationships, some of which are solved analytically, while others are calculated numerically. Particular emphasis is placed on choosing electrical and mechanical MU properties so as to achieve realistic EMG-force relationships, and physiological justification is provided for every component of the model. The model consolidates and extends several previously presented results regarding the different components of the physiological system, incorporating recent advances in understanding of physiology. In addition to combining previously isolated results in a unified model and rigorously deriving a more intuitive formulation of the EMG model of Farina and Merletti (2001), new models for several subcomponents of the muscular system have been proposed.

Firstly, a novel nonlinear input transformation is proposed which ensures that the generated muscle forces match the target force level in the isometric, undisturbed case, and which adjusts the rate-coding and recruitment characteristics of the model in a physiologically meaningful way to the force generating capabilities of the model. While previous studies (Dideriksen et al., 2010b, 2017; Contessa and De Luca, 2013; Venugopal et al., 2017) have used PID feedback control to solve this problem, we propose to separate the tasks of

1. adjusting the different model components (rate coding/recruitment and force generation) to each other, and
2. compensating for external disturbances and time-varying parameters.

While the second task inevitably must be (and physiologically is) solved using feedback control, the first one can be solved by means of a static nonlinear transformation between the desired output force level and the common drive input to the rate coding model, as proposed here. This solution entails several useful consequences. On the one hand, it allows for an analytic derivation of the rate coding scheme adjusted to the force generating capabilities of the muscle, as illustrated in Figure 2. It can be observed that the adjustment introduces physiologically reasonable modifications to the model, such as an upwards bend in the firing rate curves at high activation levels (Erim et al., 1996). This is especially interesting since the properties of sEMG-force models have been found to depend strongly on the assumed rate coding model and parameters (Keenan and Valero-Cuevas, 2007). On the other hand, from a control theoretic perspective, the introduction of this static nonlinearity represents an exact linearization of the control plant, which significantly simplifies the feedback control task as well as a subsequent analysis of the closed-loop system. Note that in this article, only the feed-forward component of the model has been considered; combining the presented model with an actual feedback controller is an obvious next step (refer to Figure 5 for an illustration of the two discussed force control schemes).

Secondly, the classical Fuglevand model (Fuglevand et al., 1993) has been extended to reflect findings regarding the dependence of MU and muscle fiber properties on MU and fiber size. In particular, not only the recruitment threshold and the peak twitch force are considered *size principle parameters* (Henneman, 1957; Henneman et al., 1965), but also the electrical conduction velocity, the time to peak force, the single fiber action potential (SFAP) amplitude and the single fiber peak twitch force. By describing the latter two quantities as a function of MU size, we have introduced an implicit measure of fiber size (fiber *diameter*) that depends on MU size. This model reflects the differences between small and large MUs and the different fiber types, while still maintaining a continuous distribution of MU and fiber properties that has been found to represent physiological findings better than discrete types with different properties (Enoka and Fuglevand, 2001; Heckman and Enoka, 2012).

Thirdly, a new model of individual MU firing rates has been proposed, which combines several desirable features: A steep, initial slope of the characteristics transitions smoothly into a flatter, linear region at higher activation levels. The onion skin principle is followed, i.e., earlier recruited MUs retain higher firing rates at all points. The initial firing rate trend, i.e., the development of the firing rates of each MU at the point of recruitment, can be freely adjusted to be either increasing or decreasing. And, finally, the convergence behavior of the firing rates of all MUs for high activation levels can also be adjusted freely. By combination with the aforementioned nonlinear input transformation, the model also displays the experimentally observed (Erim et al., 1996) increased slopes starting from the point of full recruitment. Currently, this model has not been fit to any physiological data; this is of course an obvious avenue for future work.

The described model of muscular force generation is in no way to be considered fixed: any component of the model can and should be exchanged for other models of that particular physiological subsystem, e.g., to increase or reduce model complexity, or to take future physiological insights into account. If, for example, more complex and realistic geometries are to be taken into account, the analytical, planar volume conductor and action potential propagation model used here can be exchanged against much more detailed models proposed in the literature (Lowery et al., 2004; Mordhorst et al., 2015), while still using the MU pool model, rate coding and recruitment models and force control concepts described in this article. To further increase physiological realism, regionalized MU placement could be modeled, e.g., as proposed by Robertson and Johnston (2017). Currently, effects due to the presence of muscle fatigue are not modeled; these could further be added to the model, e.g., by employing the metabolic model of Dideriksen et al. (2010a) and a force feedback loop (Dideriksen et al., 2010a; Venugopal et al., 2017). For the simulation of limb muscles, the employed model of the volume conductor should be exchanged for the cylindrical model presented by Farina et al. (2004) or a more flexible finite-element model (Lowery et al., 2004; Mordhorst et al., 2015). The restriction to isometric contractions could be resolved by implementing time-varying muscle geometry (and hence time-varying MU-electrode transmission paths), and by considering the force-length and force-velocity characteristics of skeletal muscles (Yamaguchi, 2001). And finally, an important challenge is raised by recent insights into the effect of neuromodulation on the neuromuscular system (Heckman et al., 2009), an effect that has to the authors' knowledge not been taken into account in any computational neuromuscular models so far.

The proposed model may be useful, among others, for the simulation of realistic sEMG and force signals for the validation of signal processing algorithms, for analyzing the sensitivity of the recorded signals to various physiological parameters, as well as to enhance the general understanding of physiology by evaluating the impact of model or parameter modifications on the system behavior. Preliminary versions of this model have already been used successfully for the validation of algorithms for the separation of inspiratory and expiratory activity in sEMG measurements of the respiratory muscles (Buchner et al., 2016; Petersen et al., 2017), as well as for the derivation of a novel algorithm for sEMG-based muscular activity quantification (Olbrich et al., 2018).

## Data Availability

The simulated EMG, force, and impulse train signals of all six rectus bellies, as well as the parameter values used to simulate these signals, are available for one simulated rectus abdominis muscle at the Dryad data repository (Petersen and Rostalski, 2019).

## Author Contributions

EP and PR contributed to the conception of the work. The resulting mathematical model was derived and implemented by EP under the supervision of PR. EP wrote the first draft of the manuscript. All authors contributed to manuscript revision and approved the submitted version.

### Conflict of Interest Statement

The authors declare that the research was conducted in the absence of any commercial or financial relationships that could be construed as a potential conflict of interest. Part of the research leading to the presented mathematical model was financially supported by Drägerwerk AG & Co. KGaA, Lübeck, Germany.

## Footnotes

^1^Note that – strictly speaking – the expression for *V*_*m*_(*z*) given in Equation (14) does not result in a compactly supported ψ(*z*) ∈ $C^{\infty }$, but Equation (14) could trivially be modified to comply with this requirement, e.g., by convolution with a smooth and compactly supported mollifier. As this is a purely theoretical operation with no practical relevance at all, this has not been pursued here.

^2^See previous footnote.

^3^Note that in biological tissues, the charge carriers are ions, as opposed to electrons in electric wires (Plonsey and Barr, 2007, p. 25).

## Appendix A: Proofs

Lemma 1. *The expressions given in Equations (11) and (15) to (18) are equivalent, assuming that ψ* ∈ $C^{\infty }$.

*Proof:* Expanding all terms in Equation (11) yields

(A1) $$\begin{array}{l} \hat{ı}(z,t)=\frac{\text{d}}{\text{d}z}\left[\psi \left(z-z_{i}-vt\right)H\left(z-z_{i}\right)\right]\\ \;-\frac{\text{d}}{\text{d}z}\left[\psi \left(z-z_{i}-vt\right)H\left(z-z_{i}-L_{1}\right)\right]\\ \;-\frac{\text{d}}{\text{d}z}\left[\psi \left(-z+z_{i}-vt\right)H\left(z-z_{i}+L_{2}\right)\right]\\ \;+\frac{\text{d}}{\text{d}z}\left[\psi \left(-z+z_{i}-vt\right)H\left(z-z_{i}\right)\right]. \end{array}$$

Note that the derivatives in Equation (A1) can only be understood in the sense of distributions, since the derivative of the Heaviside function *H*(*z*) can not be defined in the classical sense at *z* = 0. For some basic properties of distributions, refer to Appendix B.

Assuming ψ ∈ $C^{\infty }$ and recalling that 〈(ψ*H*)′, ζ〉 = 〈ψ′*H* + ψδ, ζ〉 (refer to Appendix B), the duality $\langle \hat{ı}\left(t\right),\zeta \rangle$ can hence be formulated as

(A2) $$\begin{array}{l} \langle \hat{ı}(t),\zeta \rangle =\langle \psi_{z_{i}+vt}^{\prime }H_{z_{i}},\zeta \rangle +\langle \psi_{z_{i}+vt}\delta_{z_{i}},\zeta \rangle \\ \;-\langle \psi_{z_{i}+vt}^{\prime }H_{z_{i}+L_{1}},\zeta \rangle -\langle \psi_{z_{i}+vt}\delta_{z_{i}+L_{1}},\zeta \rangle \\ \;+\langle {\bar{\psi }}_{z_{i}-vt}^{\prime }H_{z_{i}-L_{2}},\zeta \rangle -\langle {\bar{\psi }}_{z_{i}-vt}\delta_{z_{i}-L_{2}},\zeta \rangle \\ \;-\langle {\bar{\psi }}_{z_{i}-vt}^{\prime }H_{z_{i}},\zeta \rangle +\langle {\bar{\psi }}_{z_{i}-vt}\delta_{z_{i}},\zeta \rangle \\ \;=\langle \psi_{z_{i}+vt}^{\prime }\left(H_{z_{i}}-H_{z_{i}+L_{1}}\right)+{\bar{\psi }}_{z_{i}-vt}^{\prime }\left(H_{z_{i}-L_{2}}-H_{z_{i}}\right),\zeta \rangle \\ \;+\langle \left(\psi_{z_{i}+vt}+{\bar{\psi }}_{z_{i}-vt}\right)\delta_{z_{i}},\zeta \rangle \\ \;-\langle \psi_{z_{i}+vt}\delta_{z_{i}+L_{1}}+{\bar{\psi }}_{z_{i}-vt}\delta_{z_{i}-L_{2}},\zeta \rangle \end{array}$$

with notations $\bar{f}\left(x\right)=f\left(-x\right)$, *f*_*a*_(*x*) = *f*(*x* − *a*) and ${\bar{f}}_{a}\left(x\right)=f\left(-x+a\right)$. This is exactly equivalent to Equations (15) to (18). □

Lemma 2. *For compactly supported ψ*(*z*), *the IAP model given in Equations (15) to (18) [or equivalently, Equation (11)] yields a formulation of* $\hat{ı}\left(z,t\right)$ *that satisfies the condition (19). This also holds true if the characteristic functions* *p*_1/2_(*z*) *are replaced by smooth window functions* *w*_1/2_(*z*) *which fulfill*

(A3) $$\begin{array}{l} w_{1}\left(z_{i}+L_{1}\right)=w_{1}\left(z_{i}\right)=w_{2}\left(z_{i}\right)=w_{2}\left(z_{i}-L_{2}\right)=0 \end{array}$$

*and which yield products ψ*(*z*)*w*_1/2_(*z*) *that are differentiable with an integrable derivative*.

*Proof:* First, we consider the case of $\hat{ı}\left(z,t\right)$ as described by Equations (15) to (18), i.e., using the characteristic functions *p*_1_(*z*), *p*_2_(*z*). For compactly supported ψ(*z*),

(A4) $$\begin{array}{l} \int_{-\infty }^{\infty }\psi^{\prime }(z)\text{ d}z=0 \end{array}$$

generally holds. Furthermore,

(A5) $$\begin{array}{l} \int_{-\infty }^{\infty }\text{GEN}(t)\delta \left(z-z_{i}\right)\text{ d}z=2\psi (-vt)=2\int_{-\infty }^{-vt}\psi^{\prime }(z)\text{ d}z, \end{array}$$

(A6) $$\begin{array}{l} \int_{-\infty }^{\infty }{\text{EOF}}_{1}(t)\delta \left(z-z_{i}-L_{1}\right)\text{ d}z=-\psi \left(L_{1}-vt\right)\\ \;=-\int_{-\infty }^{L_{1}-vt}\psi^{\prime }(z)\text{ d}z, \end{array}$$

and equivalently for EOF_2_. Combining everything yields

(A7) $$\begin{array}{l} \int_{-\infty }^{\infty }\hat{ı}(z,t)\text{ d}z=2\int_{-\infty }^{-vt}\psi^{\prime }(z)\text{ d}z-\int_{-\infty }^{L_{1}-vt}\psi^{\prime }(z)\text{ d}z-\int_{-\infty }^{L_{2}-vt}\psi^{\prime }(z)\text{ d}z\\ \;+\int_{z_{i}}^{z_{i}+L_{1}}\psi^{\prime }\left(z-z_{i}-vt\right)\text{ d}z+\int_{z_{i}-L_{2}}^{z_{i}}\psi^{\prime }\left(-z+z_{i}-vt\right)\text{ d}z\\ \;=0. \end{array}$$

Secondly, considering the case of smooth window functions *w*_1/2_(*z*), we have

(A8) $$\begin{array}{l} \int_{-\infty }^{\infty }\hat{ı}(z,t)\text{ d}z=\int_{z_{i}}^{\infty }\frac{\text{d}}{\text{ d}z}\left[\psi \left(z-z_{i}-vt\right)w_{1}(z)\right]\text{ d}z\\ \;-\int_{-\infty }^{z_{i}}\frac{\text{d}}{\text{ d}z}\left[\psi \left(-z+z_{i}-vt\right)w_{2}(z)\right]\text{ d}z\\ \;=0 \end{array}$$

by the fundamental theorem of calculus if the conditions on *w*_1/2_(*z*) stated in the lemma hold. □

## Appendix B: Distributions

A distribution *T* is a continuous linear mapping *T*: *D* → ℝ, where *D* is a given set of so-called test functions. In the following, the value of a distribution *T* acting on a test function ζ shall be denoted by the duality 〈*T*, ζ〉, with test functions chosen from the set *D* of compactly supported, smooth functions. Furthermore, two basic properties of distributions are

$$\begin{array}{l} \langle \eta T,\zeta \rangle =\langle T,\eta \zeta \rangle \;\forall \eta \in C^{\infty },\zeta \in D \end{array}$$

and

$$\begin{array}{l} \langle T^{\prime },\zeta \rangle =-\langle T,\zeta^{\prime }\rangle \;\forall \zeta \in D. \end{array}$$

Moreover (following from the above), considering η ∈ $C^{\infty }$,

$$\begin{array}{l} \langle {\left(\eta H\right)}^{\prime },\zeta \rangle =-\langle \eta H,\zeta^{\prime }\rangle =-\int_{-\infty }^{\infty }(\eta H)(x)\zeta^{\prime }(x)\text{ d}x\\ \;=-{\left[\eta \zeta \right]}_{0}^{\infty }+\int_{0}^{\infty }\eta^{\prime }\zeta \text{ d}x=\langle \delta ,\eta \zeta \rangle +\langle \eta^{\prime }H,\zeta \rangle \\ \;=\langle \eta^{\prime }H+\eta \delta ,\zeta \rangle . \end{array}$$

For more background on distributions (i.e., continuous linear functionals), refer to, e.g., Clarke (2013).
